# Physiological symptoms induced by drought stress outweigh vascular pathogen infection in walnut

**DOI:** 10.1093/treephys/tpaf034

**Published:** 2025-03-26

**Authors:** Israel Jiménez Luna, Louis Santiago, Exequiel Ezcurra, MengYuan Xi, Vanessa E T M Ashworth, Eugene Nothnagel, Philippe E Rolshausen

**Affiliations:** Department of Botany and Plant Sciences, 900 University Avenue, University of California, Riverside 92521, USA; Department of Botany and Plant Sciences, 900 University Avenue, University of California, Riverside 92521, USA; Department of Botany and Plant Sciences, 900 University Avenue, University of California, Riverside 92521, USA; Department of Botany and Plant Sciences, 900 University Avenue, University of California, Riverside 92521, USA; Department of Botany and Plant Sciences, 900 University Avenue, University of California, Riverside 92521, USA; Department of Botany and Plant Sciences, 900 University Avenue, University of California, Riverside 92521, USA; Department of Botany and Plant Sciences, 900 University Avenue, University of California, Riverside 92521, USA

**Keywords:** fungal pathogens, leaf area, photosynthesis, plant hydraulics, tree

## Abstract

Drought stress can affect the success of xylem-dwelling pathogens due to modifications of the xylem environment as water potential declines. However, the interaction between these abiotic and biotic stresses on plants is complex and requires specific experiments to distinguish between multiple effects. This is especially important in agroecosystems, where monocultures of individuals facilitate disease transmission and water scarcity can lead to deficit irrigation practices to optimize water management, control canopy size and maintain crop productivity. We measured photosynthetic gas exchange, stem xylem water potential, non-structural carbohydrates (NSC), morphology and growth of walnut trees in response to two imposed stress treatments. One was the inoculation with the two cosmopolitan vascular fungal pathogens *Diplodia mutila* and *Neofusicoccum parvum*. The other was a manipulation of water availability with well-watered controls compared to deficit irrigation treatments representing 75% and 25% of well-watered controls. We found that deficit irrigation significantly reduced all measures of gas exchange and stem xylem water potential, and most morphological, growth and NSC variables. Signs of severe drought with leaf yellowing and senescing occurred at the end of the experiment when leaf water potential reached −1.6 MPa. In contrast, responses to pathogen inoculation were limited to reduced stem xylem water potential, total plant leaf area and leaf area ratio. There was no reduction in photosynthetic rate per leaf area with pathogen inoculation, but the reduction in whole plant leaf area led to an overall reduction in whole plant photosynthesis. Pathogen-induced effects were independent of the plant water status, yet they were only visible in fully irrigated trees suggesting that drought minimizes the scope of measurable symptoms. Biotic damage was not enhanced under drought stress perhaps indicating that the host had not reached a critical water stress status conducive to pathogen virulence.

## Introduction

Recent droughts have demonstrated the uncertainty of securing sufficient water for agricultural tree crops, and current models suggest that changing precipitation patterns coupled with warming temperatures will increase drought stress in many regions of the globe (Jump et al. 2017, Naumann et al. 2018). In natural systems, tree mortality (Allen et al. 2010) is driven by hydraulic failure and carbon starvation under drought (McDowell et al. 2008, McDowell et al. 2013), making this a useful framework for addressing mortality mechanisms in other plant systems. However, in agricultural systems, trees are under a different set of constraints than in natural systems, because they are irrigated to maintain high yield and quality crops. Deploying a deficit irrigation strategy, which involves minimizing irrigation outside of the drought-sensitive growth stages of a crop, has become instrumental to optimize water management, control canopy size and maintain crop productivity (Chai et al. 2016, Galindo et al. 2018).

Drought stress is often thought to predispose trees to diseases (Farooq et al. 2009, Whyte et al. 2016). Within the framework of the ‘disease triangle’, which describes interactions between a pathogen and plant host as affected by weather (Scholthof 2007, Leisner et al. 2023), drought can affect the pathogen and the host in different ways, leading to a positive or negative shift in disease outcomes. A review of pathogen-drought studies demonstrated that direct effects of drought on pathogens are generally negative but does not always result in higher biotic damage. Outcomes can be predicted by the type of trophic interaction, the host tissue type and the water stress severity (Jactel et al. 2012, Oliva et al. 2014). The interaction between drought stress and vulnerability to disease is particularly complex for vascular pathogens. The host responds to infection by compartmentalization, with different anatomical boundaries being laid out strategically to spatially reinforce cell walls and limit movement of the pathogen as described by the Compartmentalization Of Decay in Trees (CODIT) model (Shigo and Marx 1977, Pearce 1996). This response leads to xylem occlusion and results in a loss of hydraulic function of host xylem vessels (Pouzoulet et al. 2019), which may indirectly affect the host physiology (Desprez-Loustau et al. 2006). Under severe water-stress, a cumulative effect on the host water transport induced by pathogen occlusion and drought cavitation can result in increased tree mortality in both forest (Schoeneweiss 1983, Appel 1984, Mullen et al. 1991, Desprez-Loustau et al. 2006) and fruit (Galarneau et al. 2019, Agusti-Brisach et al. 2020, Dong et al. 2021) trees. However, under mild water stress conditions, trees can be less damaged by wood endophytic fungal pathogens than unstressed trees (Jactel et al. 2012). Studies investigating how the duration and severity of deficit irrigation influence the balance of host-pathogen interactions in a tree crop have remained limited in scope.

Here, we study the physiological response of English walnut (*Juglans regia* L.) to the combination of water stress and vascular pathogens. *Neofusicoccum parvum* and *Diplodia mutila* are two taxa in the family Botryosphaeriaceae that are widely distributed and affect several hosts causing symptoms of wood decay, canker and dieback (Phillips et al. 2013, Silva-Valderrama et al. 2024). Both fungi have also been reported to have a commensal endophytic life stage that turns pathogenic when the host is under stress (Slippers and Wingfield 2007). Diseases caused by these fungi are common in many countries producing walnut, although *N. parvum* is described to be more virulent than *D. mutila* (Jimenez Luna et al. 2022). *Juglans* species have been well characterized for their hydraulic physiology, especially with respect to water stress responses (Tyree et al. 1993, Cochard et al. 2002, Sun et al. 2011, Liu et al. 2019). English walnut is grown globally and is valued for its lumber and nut fruit. California walnut production is mostly concentrated in semi-arid zones where temperature and moisture are already approaching biophysical thresholds. The increasing temperature stress and number of dry days, combined with highly variable seasonal rainfall and a decline in winter chill hours have been projected to negatively affect yields of several deciduous tree crops, especially walnut (Lobell et al. 2006, Lee and Sumner 2016). Walnut is sensitive to water deficits as suggested by its stomatal sensitivity to humidity as well as drought-induced leaf abscission habit (Parker and Pallardy 1985, Pallardy and Rhoads 1993, Tyree et al. 1993, Cochard et al. 2002, Sun et al. 2011). This study focuses on the biotic and abiotic stress interaction and tests the hypothesis that deficit irrigation would exacerbate biotic damage in walnut trees. We measured the physiological and growth responses of walnut to two levels of water limitation and determined if those were altered by infection with vascular pathogens. This research provides insightful information on disease outcomes in response to deficit irrigation in an agricultural cropping system and a reference framework on how the adoption of cultural practices in response to climate change could affect walnut tree health.

## Materials and methods

### Plant material

The experiment was conducted outdoors, in a shade house at University of California, Riverside (UCR). Dormant 2-year-old English walnut (*J. regia*) trees cv. ‘Paradox’ were planted into 9 L pots filled with clay loam soil (UCR Soil Mixture). At the onset of the experiment, the selected trees had uniform phenotypes with no leaves because trees were dormant and averaged 40.4 ± 1.2 cm in height and 18.91 ± 0.23 mm in diameter at the base of the tree, 3 cm above the soil line. The study consisted of nine treatments (3 levels of watering regimes × 3 types of inoculation) with 10 trees per treatment (90 trees total) set in a completely randomized design. The experiment started on 11 February 2020 (Julian Day 42) and lasted 23 weeks until 20 July 2020 (Julian Day 201). Deficit irrigation and pathogen inoculation were initiated on the same day on dormant trees. Each tree was fertilized with 10 g of 14-14-14 N-P-K slow-release fertilizer (Osmocote, Scotts Co., Marysville, Ohio, USA) in March before budbreak.

### Deficit irrigation treatments

To establish the deficit irrigation treatments, water deficit was first calculated on an additional pilot set of 10 dormant walnut trees of similar size, height and age prior to the onset of the main experiment. Soil water holding capacity was determine by weight. Water loss from pots via transpiration and soil evaporation was quantified during the experiment by watering trees and allowing them to drain for 1 hour and then weighed. After 24 hours, pots were weighed again before watering. Field capacity was then calculated as wet weight minus dry weight divided by dry weight. The 100% field capacity mean was calculated for all 10 potted walnut trees and the deficit treatments received 75 and 25% of this amount (Edwards et al. 2007, Sosnowski et al. 2011). The same process was performed every 6 weeks to determine field capacity as time, tree phenotypic characteristics and environmental conditions changed. Furthermore, volumetric soil water content was measured using a soil moisture sensor (Hydrosense II, Campbell Scientific, Australia) between 0700 and 0800 hours once per week for all 23 weeks to monitor water content in the soil. The 20 cm sensor was inserted next to the trunk inside of each pot. The tree water treatments were guided for the first 12 weeks of the experiment by the soil water holding capacity as determined by the soil weighing method and readings from the soil water sensor. Once leaves were fully developed predawn (Ψ_PD_) and midday stem (Ψ_MD_) water potential were also recorded (on Julian Day 125) to maintain the irrigation regimes within a range of the tree water stress. The target range were determined based on Ψ_MD_ according to Fulton and Buchner (2015) with 100% water content equaled −0.4 to −0.8 MPa (control), 75% water content equaled −0.6 to −1 MPa (mild, 25% deficit irrigation); and 25% water content ≤1.2 MPa (severe, 75% deficit irrigation). All 90 walnut trees used in the main experiment were equally divided in these three irrigation groups (30 trees/group).

### Pathogen treatments

Within each deficit irrigation group, 10 trees were inoculated with either *N. parvum* strain UCR-NP2 or *D. mutila* strain UCR-DM1 or were mock-inoculated in the case of control trees. Inoculation of trees was performed according to Jiménez Luna et al. (2022). Pure cultures of *N*. *parvum* and *D*. *mutila* were grown on potato dextrose agar (PDA) media at 25 °C for 5 days. In February 2020, when trees were dormant, a 5 mm diameter wound was made on the trunk to cambium depth at ~5 cm above soil level using a cork borer. Trees were inoculated with 5 mm plugs of *N*. *parvum* and *D*. *mutila* cultures with mycelia facing the wound and sealed with parafilm. Control plants were inoculated with sterile agar mycelium plugs. At the end of the experiment, wood necrotic lesion length was measured after shaving the tree bark with a sterile blade. To confirm that symptoms were caused by the pathogens, three trees per treatment were selected randomly and wood samples were collected from necrotic wood. Wood samples were surface sterilized in 1% sodium hypochlorite (pH 7.2) for 30 seconds and washed in water for 1 min three times. Wood chips were plated on PDA medium and incubated for a week at room temperature to confirm the recovery of the pathogen (Jiménez Luna et al. 2022).

### Photosynthesis measurements

Once leaves reached maturity, maximum light-saturated net CO_2_ assimilation rate (*A*_max_; Table 1), transpiration (*E*) and stomatal conductance (*g_s_*) were measured on the same leaf using a portable Li-Cor LI-6800 photosynthesis machine (Li-Cor, Inc. Lincoln, NE, USA) with CO_2_ at 400 ppm, fan speed at 8000 rpm, 25 *°*C temperature, 50% *relative humidity, 1.0 kPa vapor pressure deficit and* ambient saturating light. Measurements were conducted once per week between 0700 and 1100 hours from 17 May (Julian Day 138) to 19 July (Julian Day 201) 2020, for all treatments. Five trees were selected randomly from each treatment and three fully developed intact leaves of homogeneous size were selected for each tree to measure gas exchange. Whole plant photosynthesis (*A*_plant_) was calculated by multiplying the photosynthetic rate by total plant leaf area (LA_plant_; see below). Once per week, maximum quantum efficiency of PSII (*F_v_*/*F_m_*) was measured using LI-6800 between midnight and 0100 hour on the same day as gas exchange measurements using a single dark-adapted leaf per tree, for a total of five random trees within each treatment.

**Table 1:** Variables and parameters measured in this study shown with symbols and units.

Response variable	Symbol	Units
**Photosynthesis measurements**		
Maximum photosynthetic net CO_2_ assimilation rate	*A*_max_	μmol m^−2^ s^−1^
Transpiration rate	*E*	mol m^−2^ s^−1^
Stomatal conductance to water vapor	*g*_s_	mol m^−2^ s^−1^
Whole-plant photosynthesis	*A*_plant_	μmol plant^−1^ s^−1^
Maximum quantum efficiency of PSII	*F*_v_/*F*_m_	Relative units
**Water relations**		
Predawn stem xylem water potential	Ψ_PD_	MPa
Midday stem xylem water potential	Ψ_MD_	MPa
Water potential difference	ΔΨ	MPa
**Tree growth/morphology**		
Specific leaf area	SLA	cm^2^ g^−1^
Total plant leaf area	LA	m^2^
Leaf area ratio	LAR	cm^2^ g^−1^
Relative growth rate of leaf number	*RGR*_L_	no no^−1^ day^−1^
Relative growth rate of height	*RGR*_H_	cm cm^−1^ day^−1^
Relative growth rate of stem	*RGR*_D_	mm mm^−1^ day^−1^
Leaf dry mass	*M*_L_	g
Stem dry mass	*M*_S_	g
Root dry mass	*M*_R_	g
Total plant dry mass	*M*_P_	g
Root-to-shoot ratio	R/S	g g^−1^
**Non-structural carbohydrates**		
Soluble carbohydrates	SC	μg mg^−1^
Digested starch	DS	μg mg^−1^
**Pathogen virulence**		
Wood necrotic lesion	NL	mm

### Water potential

Stem xylem water potential was measured biweekly between 4 May (Julian Day 125) and 13 July (Julian Day 195) 2020 using a Scholander Pressure Chamber (Deloire and Heyns 2011). Fully expanded terminal leaflets were randomly selected, and predawn water potential (Ψ_PD_) was measured between 0100 and 0400 hours and mid-day water potential (Ψ_MD_) was measured between 1100 and 1300 hours. Measurements were conducted on four random trees per treatment. For measurements, two fully developed leaves in the upper canopy were covered with a Whirl-Pak bag and foil 8 hours prior to measurement to allow tissue to equilibrate with stem xylem. Leaves were excised from the petiole using a razor blade and inserted into the pressure chamber seals. The chamber was pressurized slowly, and pressure was recorded when a water meniscus began to form on the cut petiole surface (Knipfer et al. 2018). We also calculated the delta between Ψ_PD_ and Ψ_MD_ (ΔΨ) as a measure of the diurnal range of stem xylem water potential.

### Tree morphology and growth

The total amount of leaves, stem diameter and tree height were recorded for every tree at the end of the experiment. Stem diameter was measured 3 cm above the soil line between 0800 and 1000 hours using a digital caliper (Fisher Scientific, Pittsburgh, PA). Stem height was also measured from the soil surface to the apical meristem using a tape measure. Leaf area for every leaf was measured by using an area meter (Li 3000A, Li-Cor). Four walnut trees per treatment were selected for biomass measurements. All leaves were removed from the stem using pruning shears, wrapped in foil and placed in an oven for 48 hours at 65 °C. The root system was removed from the pot and washed in a sieve to remove adherent soil. After the root system was dry, it was placed in foil and dried for 48 hours at 65 °C. Using a digital balance, dry weights for leaves, stems and roots were measured and recorded. Specific leaf area (SLA, cm^2^ g^−1^) was calculated as the ratio of leaf area per leaf dry mass excluding the petiole. Whole plant leaf area (LA_plant_; m^2^ plant^−1^) was calculated by multiplying leaf area × number of leaves per plant. Leaf area ratio (LAR; cm^2^ leaf g^−1^ plant) was calculated as total plant leaf area per total plant biomass. Root to shoot ratio (R/S; g root g^−1^ shoot) was calculated as total below-ground mass divided by the total above-ground mass of the plant (Santiago et al. 2012). We used relative growth rate (RGR) to account for slight differences in plant size. Relative growth rate of height (RGR_H_; cm cm^−1^ × month) was calculated as (lnH_1_ –lnH_0_)/(t_1_-t_0_) where H_0_ and H_1_ were initial and final heights (cm) and t_1_-t_0_ was the time period (11 February–20 July 20). A similar equation was used to estimate relative growth rate of leaf count (RGR_L_) (Santiago et al. 2012), and relative growth rate of basal trunk diameter (RGR_d_).

### Non-structural carbohydrates

Non-structural carbohydrates (NSC) were measured in wounded/inoculated and healthy woody xylem for two irrigation regimes (100 and 25% deficit irrigation treatments). For each treatment, 4 cm long trunk sections at the point of inoculation (2 cm above and below the wound) were collected from three random trees at the end of the experiment. The bark was peeled off the trunk and the wood tissues were further separated in two groups. One group included sections of the trunk samples that were wounded and inoculated and that showed necrotic lesions when infected with pathogens and a reaction zone for the mock inoculated trees. The other group contained sections of the xylem tissues on the opposite side of the point of inoculation that did not show necrotic lesions. Methods for NSC analyses from walnut wood and bark were adapted from Loescher and Nevins (1972) and Tixier et al. (2020). Dried wood samples were ground into fine, homogeneous powders using a MM400 grinder (Retsch, Haan, Germany). To extract soluble carbohydrates (SC), 25 mg of dry wood powder samples were loaded into a 2 mL conical polypropylene snap cap tube, one mL of distilled water was added, and the mixture was vortexed. The tube was incubated in a dry-block heater at 70 °C for 15 min with vortexing every 3 min, and then centrifuged for 10 min at 21,000 g. A 500 μL aliquot was pipetted from the top of the SC supernatant and transferred to a new 1.7 mL conical polypropylene tube, and stored overnight at −20 °C. The remaining supernatant above the pellet was discarded and the pellet was washed twice with 1 mL of 80% ethanol, by vortexing, centrifuging 10 min at 21,000 g, and discarding the supernatant. The washed pellet was resuspended in 0.5 mL of MOPS buffer [30 mM 3-(N-morpholino) propanesulfonic acid, 3 mM CaCl_2_, adjusted to pH 7.0 with KOH]. For digestion of starch, a 0.5 mL aliquot containing 200 activity units of α-amylase from porcine pancreas (200 activity units; Sigma-Aldrich Type I-A, diluted with MOPS buffer) was added to the 0.5 mL of washed wood pellet and placed on a 250 RPM shaker at 37 °C (Huber and Nevins 1977). After 20 hours of incubation, the digested materials were centrifuged for 10 min at 21,000 g. A 500 μL aliquot was pipetted from the top of the digested starch (DS) supernatant and transferred to a new 1.7 mL conical polypropylene tube. Dilutions (1:9, v/v) of both the thawed SC and DS supernatants were prepared by mixing 100 μL of the supernatant with 900 μL of distilled water in a new 1.7 mL conical polypropylene tube. Carbohydrate contents of the SC and DS supernatants were determined by the phenol-sulfuric acid assay as described by Ashwell (1966), except all volumes were reduced to 25% of the stated volumes. For the SC supernatants, 200 μL of the (1:9, v/v) diluted SC supernatant and 300 μL of distilled water were first added to a glass tube (13 × 100 mm). For the DS supernatants, 80 μL of the (1:9, v/v) diluted DS supernatant and 420 μL of distilled water were added to a glass tube. A 12.5 μL aliquot of 80% (w/w) phenol was pipetted into each tube, which was then vortexed for 5 s. A 1.25 mL aliquot of H_2_SO_4_ (96% w/w) was rapidly added by re-pipetting into each tube, which was then vortexed for no more than 3 s. After allowing the tubes to cool at room temperature for 30 min, a spectrophotometer was used to measure absorbance at 485 nm against a blank prepared by using 500 μL of distilled water. Standard curves were prepared using 5, 10, 20, 30, 40 and 50 μg amounts of D-glucose or maltose monohydrate. Least squares linear regression gave *y* = 0.0231 + 0.0398*x* with R^2^ = 0.9959 where *y* = A485 and *x* = μg D-glucose, or *y* = 0.0106 + 0.0404*x* with R^2^ = 0.9976 where *y* = A485 and *x* = μg maltose monohydrate. The near identity of these equations is consistent with the anticipated nearly complete hydrolysis of maltose to two glucoses in the phenol-sulfuric acid assay.

### Statistical analysis

Normality and homogeneity of variances were evaluated using normality Q-Q plots and Levene’s test and data were log transformed where needed. For the main factors ‘pathogen’ and ‘water treatment’, the ‘lem4’ package in R studio was used to determine *P* values for each parameter (Table 1). Pathogen and irrigation treatments and their interactions were considered as fixed effects in all analyses. In repeated measures ANOVA, the tree identity (experimental replication) was added as a random effect. For significant effects (*P* < 0.05) between pathogen and water treatment interaction, means were followed by Tukey’s test. An ANOVA was also used to determine the effects of water treatment (100, 75 and 25% of water holding at field capacity) on stem water potential using the previous steps described above, but with time treated as a repeated measure since leaves were collected from the same trees six times throughout the experiment. Furthermore, ANOVAs followed by a post-hoc Tukey HSD were used to determine the combined effects of inoculation and water treatments on *A*_plant_, LA_plant_, LAR, RGR_L_ and NSC in bark and wood tissues using data from the last date of the experiment (Julian Day 201). Open-source statistical computing language *R* version 3.4.1 (Team 2020) was used for all analyses.

## Results

**Figure 1:**
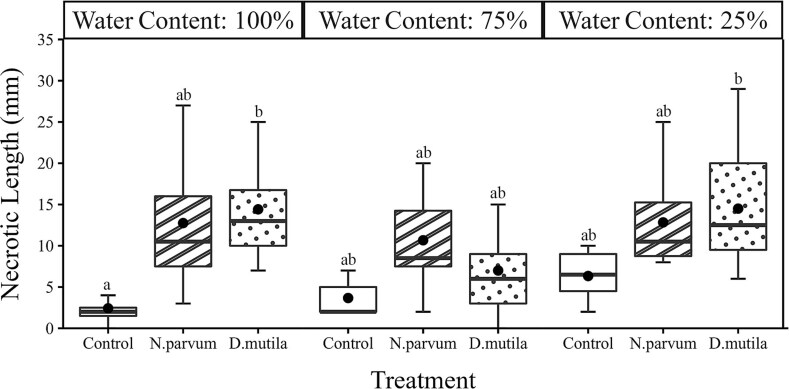
Box and whisker charts showing variation of wood necrotic length in *J. regia* L. mock-inoculated control (blank boxplot), *D. mutila*-inoculated (dotted boxplot) and *N. parvum-*inoculated (stripe boxplot) trees under 100, 75 and 25% water content. The upper and the lower edges of each box indicate the 75th and 25th percentiles, respectively. Large black dots inside each box plot represent the mean; the horizontal line within each box is the median. Different letters indicate statistically significant differences (Tukey HSD test *P* < 0.05).

Our results showed that within the experimental timeframe, deficit irrigation significantly impacted a wide range of biological processes in walnut trees (Tables 1–3). Trees under severe deficit irrigation suffer leaf yellowing and abscission at the end of the experiment regardless of the pathogen (Julian Day 180). In contrast, the effects of pathogen inoculation were limited in range and affected similarly Ψ_PD_, Ψ_MD_, LA, LAR and *A*_plant_ for both *N. parvum* and *D. mutila* (Tables 1–3). Noticeable differences between the two pathogens were measured on the interactions between pathogen with irrigation treatments whereby *D. mutila* displayed a marginal yet significant effect on *F_v_*/*F_m_*, *A*_plant_ and LAR (*P* < 0.05) whereas and *N. parvum* showed a highly significant effect on LAR (*P* < 0.001) and *M*_R_ (*P* < 0.05)_._ As expected, pathogen treatment significantly affected NL, albeit independently of the host water status (Tables 2 and 3). NL were only significantly longer than the mock inoculated controls for *D. mutila* in well-watered trees (Figure 1).

**Table 2:** Results of repeated-measures analysis of variance for gas exchange, water relations, physiological and morphological traits, NSC and pathogen growth on *J. regia* L. under 100, 75 and 25% water content inoculated with *D. mutila* for 23 weeks post-inoculation (February–July 2020)

Parameters		Pathogen			Water			Pathogen × water		
	df (resid)	*F*	*P*	Sig.	*F*	*P*	Sig.	*F*	*P*	Sig.
**Photosynthesis measurements**										
*A*_max_	6	0.67	4.43E-01	–	57.65	1.21E-04	***	0.27	7.71E-01	–
*E*	6	0.86	3.90E-01	–	181.20	4.32E-06	***	3.82	8.51E-02	–
*g*_s_	17	0.12	7.32E-01	–	40.57	3.37E-07	***	0.29	7.51E-01	–
*F_v_*/*F_m_*	27	1.50	2.31E-01	–	21.00	9.35E-05	***	5.03	3.34E-02	*
*A*_plant_	18	15.02	1.11E-04	**	50.65	4.05E-08	***	4.25	3.09E-02	*
**Water relations/hydraulics**										
Ψ_PD_	41	10.56	2.31E-03	**	25.51	9.53E-06	***	0.03	8.74E-01	–
Ψ_MD_	41	16.62	2.04E-04	***	34.50	6.54E-07	***	2.17	1.48E-01	–
ΔΨ	41	3.93	5.43E-02	–	7.20	1.05E-02	**	1.41	2.42E-01	–
**Tree growth/morphology**										
LA	18	12.00	2.77E-03	**	29.71	1.99E-06	***	2.94	7.86E-02	–
SLA	20	0.05	8.21E-01	–	1.45	2.42E-01	–	0.29	6.00E-01	–
R/S	20	4.60	4.45E-02	*	3.33	8.28E-02	–	0.33	5.70E-01	–
LAR	20	18.13	4.74E-04	***	42.83	1.44E-07	***	4.58	2.46E-02	*
* RGR*_H_	66	0.53	4.69E-01	–	2.09	1.31E-01	–	0.93	4.00E-01	–
*RGR*_L_	66	0.17	6.85E-01	–	30.94	3.31E-10	***	1.40	2.54E-01	–
*RGR*_D_	68	0.64	4.26E-01	–	0.00	2.47E-03	**	0.43	4.34E-01	–
*M*_S_	20	1.18	2.90E-01	–	3.33	8.31E-02	–	0.01	9.19E-01	–
*M*_L_	20	0.00	9.69E-01	–	44.69	1.66E-06	***	3.40	8.01E-02	–
*M*_R_	20	0.79	3.84E-01	–	0.96	3.39E-01	–	0.48	4.79E-01	–
*M*_P_	18	0.08	7.81E-01	–	1.81	1.92E-01	–	0.19	8.28E-01	–
**Non-structural carbohydrates**										
SC unwounded wood	8	2.38	1.62E-01	–	26.39	8.88E-04	***	5.04	5.50E-02	–
DS unwounded wood	8	0.23	6.47E-01	–	0.40	5.43E-01	–	0.44	5.26E-01	–
SC wounded/inoculated wood	8	1.40	2.71E-01	–	3.51	9.80E-02	–	0.00	9.91E-01	–
DS wounded/inoculated wood	8	3.29	1.07E-01	–	0.06	8.21E-01	–	0.07	7.92E-01	–
**Pathogen virulence**										
NL	66	17.84	7.54E-05	***	0.33	7.18E-01	–	0.23	7.92E-01	–

Bold stars indicate statistically significant differences among means (*** = *P* ≤ 0.001; ** = *P* ≤ 0.01; * = *P* ≤ 0.05).

**Table 3:** Results of repeated-measures analysis of variance for gas exchange, water relations, physiological and morphological traits, NSC and pathogen growth on *J. regia* L. under 100, 75 and 25% water content inoculated with *N*. *parvum* for 23 weeks post-inoculation (February–July 2020)

Parameters		Pathogen			Water			Pathogen × water		
	df (resid)	*F*	*P*	Sig.	*F*	*P*	Sig.	*F*	*P*	Sig.
**Photosynthesis measurements**										
*A*_max_	5	1.77	2.62E-01	–	96.90	6.67E-04	***	0.11	9.16E-01	–
*E*	5	0.08	7.90E-01	–	28.17	1.90E-03	**	0.43	6.74E-01	–
*g*_s_	17	0.08	7.76E-01	–	39.09	4.38E-07	***	0.12	8.92E-01	–
*F_v_*/*F_m_*	27	0.77	3.87E-01	–	14.35	7.73E-04	***	0.96	3.37E-01	–
*A*_plant_	18	11.38	3.38E-04	**	44.96	9.99E-08	***	3.03	7.36E-02	–
**Water relations/hydraulics**										
Ψ_PD_	43	24.36	1.25E-05	***	15.66	2.79E-04	***	2.84	1.00E+00	–
Ψ_MD_	43	17.70	1.29E-04	***	29.19	2.68E-06	***	3.82	5.71E-02	–
ΔΨ	43	1.13	2.94E-01	–	7.74	8.00E-03	**	0.85	3.61E-01	–
**Tree growth/morphology**										
LA	18	9.07	7.49E-03	**	41.93	1.68E-07	***	1.87	1.83E-01	–
SLA	20	0.11	7.40E-01	–	0.19	6.70E-01	–	0.13	7.25E-01	–
R/S	20	1.29	2.70E-01	–	0.29	5.99E-01	–	2.15	1.58E-01	–
LAR	20	31.66	2.44E-05	***	50.77	3.98E-08	***	11.25	6.78E-04	***
*RGR*_H_	66	0.07	7.91E-01	–	1.61	2.07E-01	–	0.13	8.79E-01	–
*RGR*_L_	66	1.08	3.02E-01	–	33.20	1.07E-10	***	1.18	3.15E-01	–
*RGR*_D_	68	0.11	7.38E-01	–	7.84	6.64E-03	**	0.72	3.98E-01	–
*M*_S_	20	0.19	6.67E-01	–	9.30	6.32E-03	**	1.90	1.83E-01	–
*M*_L_	20	0.43	5.19E-01	–	61.54	1.58E-07	**	3.80	6.55E-02	–
*M*_R_	20	2.21	1.53E-01	–	7.14	1.47E-02	*	5.87	2.51E-02	*
*M*_P_	18	1.62	2.19E-01	–	6.49	7.55E-03	**	2.92	7.96E-02	–
**Non-structural carbohydrates**										
SC unwounded wood	8	0.93	3.63E-01	–	14.35	5.33E-03	**	0.95	3.58E-01	–
DS unwounded wood	8	5.31	5.00E-02	*	2.75	1.36E-01	–	2.68	1.40E-01	–
SC wounded/inoculated wood	8	1.62	2.39E-01	–	2.56	1.49E-01	–	0.94	3.62E-01	–
DS wounded/inoculated wood	8	1.92	2.04E-01	–	4.17	7.54E-02	–	2.17	1.79E-01	–
**Pathogen virulence**										
NL	66	46.15	3.77E-09	***	1.63	2.04E-01	–	1.06	3.53E-01	–

Bold stars indicate statistically significant differences among means (*** = *P* ≤ 0.001; ** = *P* ≤ 0.01; * = *P* ≤ 0.05).

**Figure 2:**
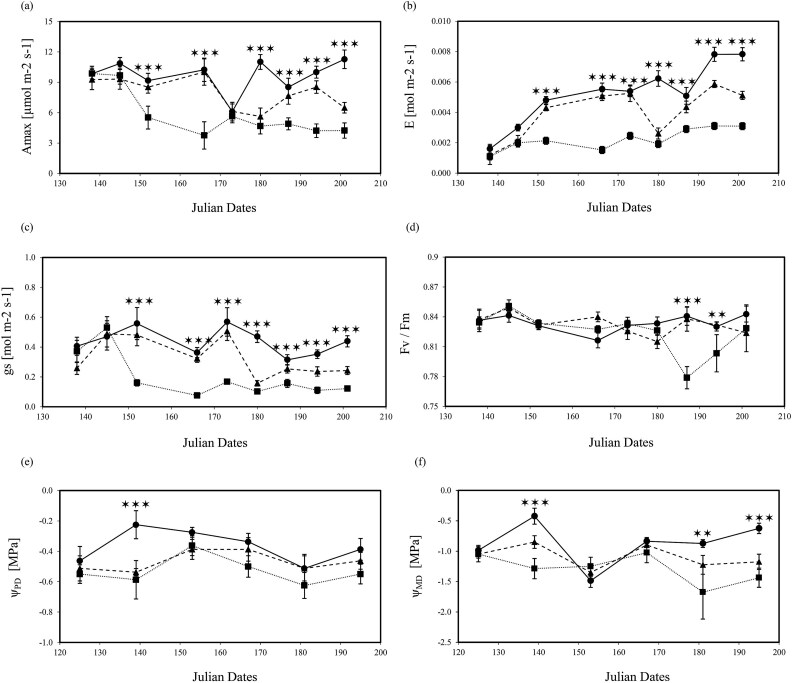
Measurements of (a) maximum photosynthetic CO_2_ assimilation rate (A_max_), (b) transpiration rate (E), (c) stomatal conductance (g_s_), (d) maximum quantum efficiency of PSII (*F*_v_/*F*_m_), (e) predawn (Ψ_PD_) and (f) midday (Ψ_MD_) water potential in non-inoculated *J. regia* L. trees. Data were collected for all three irrigation treatments: 100% (•) solid line, 75% (▲) dash line, 25% (■) dotted line water content for 11 weeks. Each point is the mean ± SE of *n* = 5 trees. Asterisks show levels of significance (Tukey HSD test, *** = *P* ≤ 0.001; ** = *P* ≤ 0.01).

Deficit irrigation severely affected plant gas exchange and photosynthesis *A*_max_ (*P* < 0.001), *E* (*P* < 0.01), *g*_s_ (*P* < 0.001) and *F*_v_/*F*_m_ (*P* < 0.001) regardless of the fungal infection (Tables 2 and 3). In the severe 75% deficit irrigation treatment, reduced *A*_max_, *E* and *g*_s_ occurred on Julian Day 152 (110 days post-deficit irrigation), whereas under the milder 25% deficit irrigation treatment, effects became apparent at Julian Day 180 (Figure 2). Once the effects of deficit irrigation on gas exchange occurred, they were sustained until the end of the experiment. A marginal negative effect of severe deficit irrigation on *F*_v_/*F*_m_ (*P* < 0.05) was only measured toward the end of the experiment on Julian Day 188 (Table 2; Figure 1). The pathogen had no effects on leaf-level gas exchange parameters.

**Figure 3:**
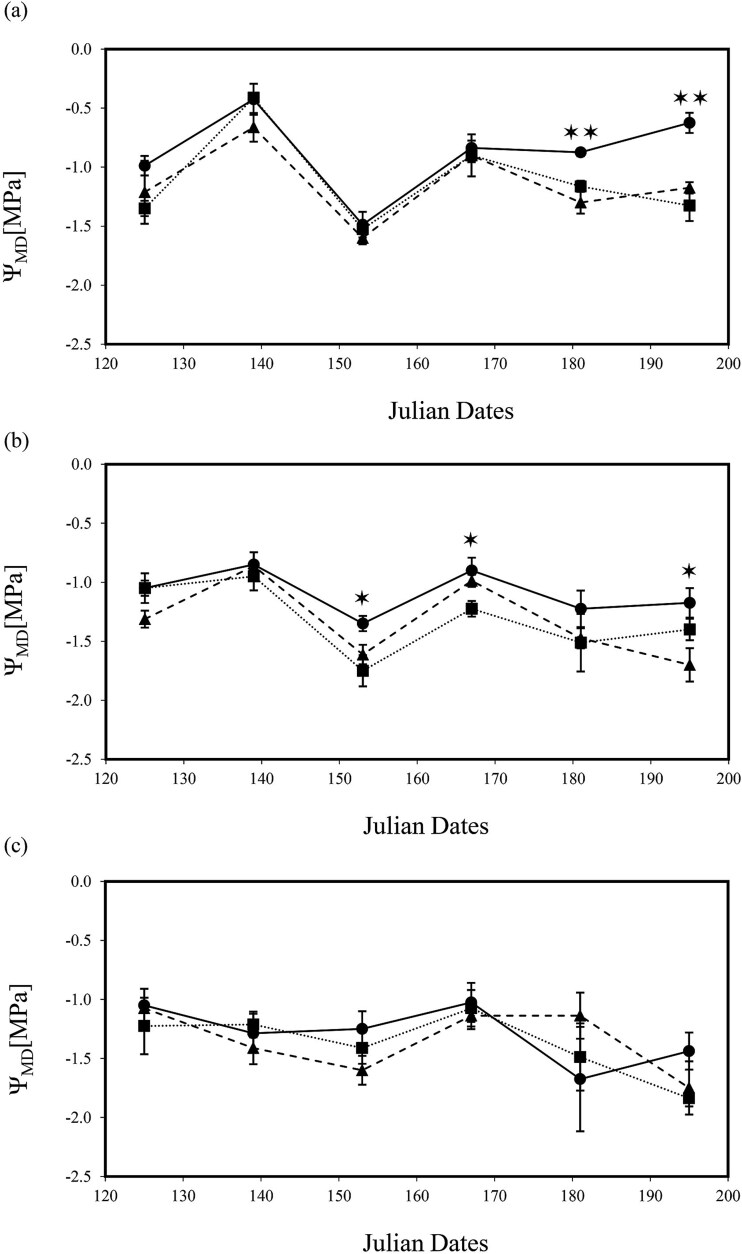
Stem midday leaf water potential (Ψ_MD_) measurements for *J. regia* L. trees under (a) 100%, (b) 75% and (c) 25% water content. Data were collected between May and July 2020 for 10 weeks for all inoculation treatments: mock (•) solid line, *N. parvum* (▲) dash line, *D. mutila* (■) dotted line. Asterisks show levels of significance (Tukey HSD test, ** = *P* ≤ 0.01; * = *P* ≤ 0.05). Each point is the mean ± SE of *n* = 4 trees/group.

**Figure 4:**
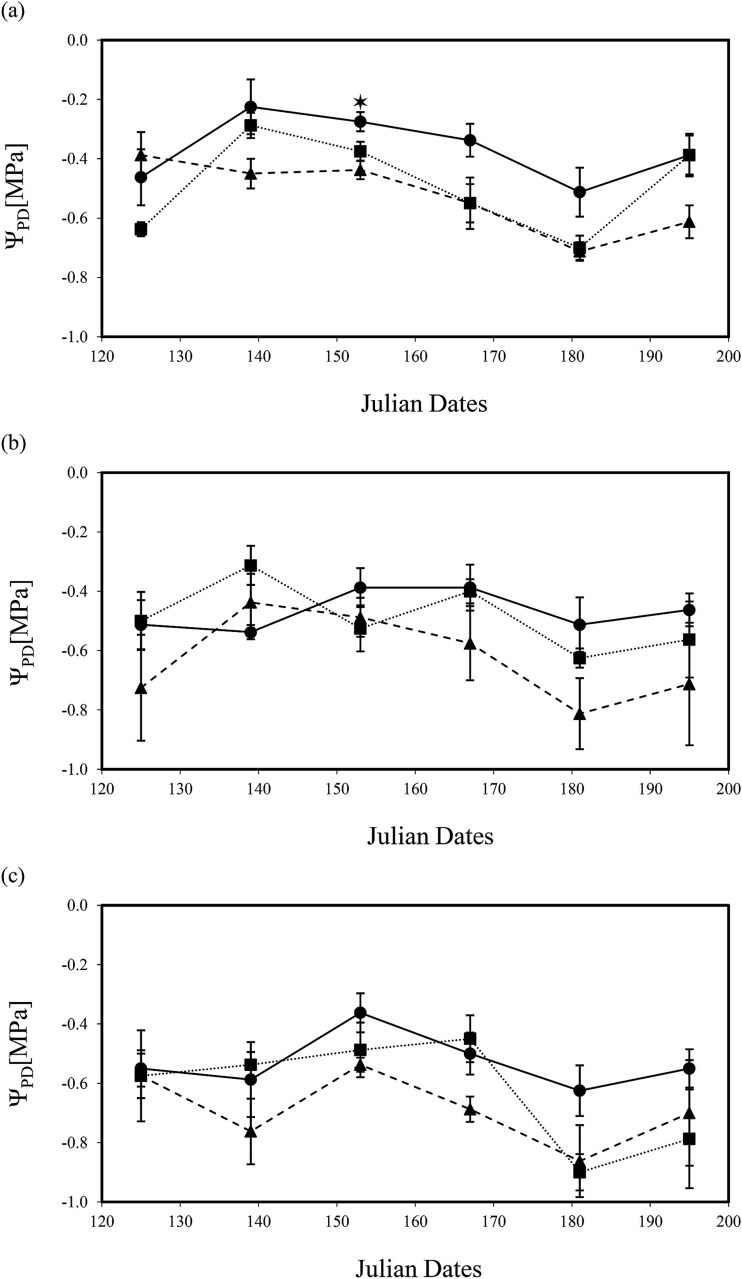
Stem predawn leaf water potential (Ψ_PD_) measurements for *J. regia* L. trees under (a) 100%, (b) 75% and (c) 25% water content. Data were collected for 10 weeks between May and July 2020 for all inoculation treatments: mock (•) solid line, *N. parvum* (▲) dash line, *D. mutila* (■) dotted line. Asterisks show levels of significance (Tukey HSD test, ** = *P* ≤ 0.01; * = *P* ≤ 0.05). Each point is the mean ± SE of *n* = 4 trees/group.

Leaf water potential measurements showed that trees were not consistently maintained within the desired Ψ_MD_ target range values (Figure 2f) for 100% irrigation (i.e., control treatment −0.4 to −0.8 MPa), 25% deficit irrigation (−0.6 to −1 MPa) and 75% deficit irrigation (≤ −1.2 MPa). Values for Ψ_PD_ and Ψ_MD_ were affected by both deficit irrigation and pathogen infection although independently of one another (Tables 2 and 3). Effects of irrigation treatments on trees Ψ_MD_ were mainly significantly observed after Julian Day 182 (*P* < 0.01; Figure 2f). However, the recorded Ψ_PD_ values (Figure 2e) indicated that trees were able to recharge their water status at night since no discernable effects were measured across treatments. Reduced water potential with pathogen inoculation was only seen in Ψ_MD_ in trees under full irrigation (*P* < 0.01; Figure 3a) toward the end of the experiment on Julian Day 182 (140 days post treatment) when trees reached Ψ_MD_ -1.6 MPa. In contrast, effects of pathogen inoculation were marginal (*P* < 0.05; Figure 3b) or non-significant (Figure 3c) under mild or severe deficit irrigation conditions, respectively. In addition, infection with pathogens showed marginal effects in Ψ_PD_ of well watered trees (*P* < 0.05; Figure 4a) or no significant effect under mild or severe deficit irrigation (Figure 4b and 4c).

**Figure 5:**
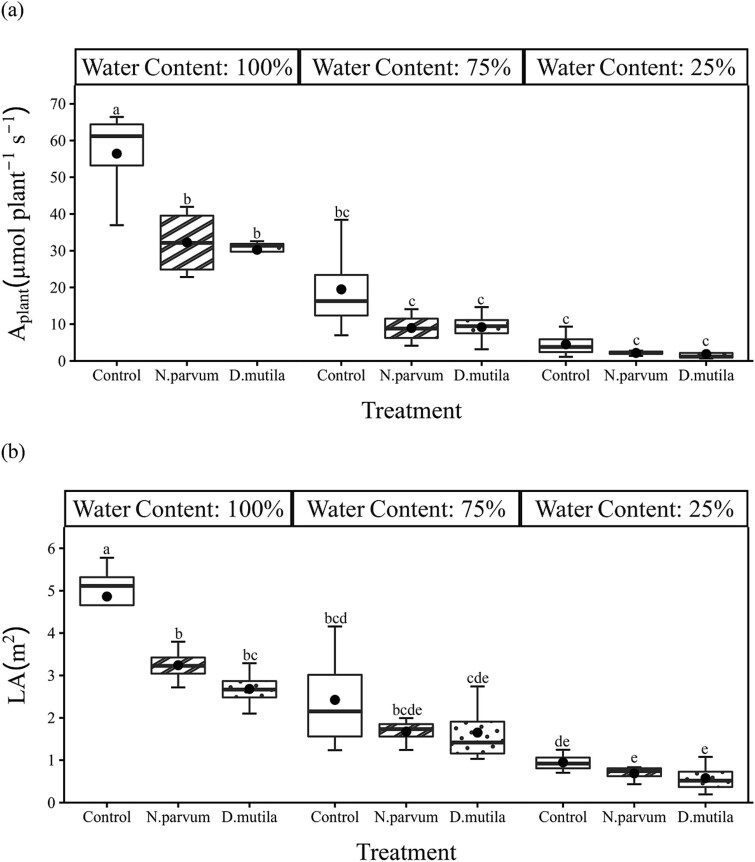
Box and whisker charts showing variation of (a) whole plant photosynthesis (A_plant_) and (b) total plant leaf area (LA) in *J. regia* L. mock-inoculated control (blank boxplot), *N. parvum*-inoculated (stripe boxplot) and *D. mutila*-inoculated (dotted boxplot) trees under 100, 75 and 25% water content treatments. The upper and the lower edges of each box indicate the 75th and 25th percentiles, respectively. Large black dots inside each box plot represent the mean and the horizontal line within each box is the median. Different letters indicate statistically significant differences (Tukey HSD test *P* < 0.05).

**Figure 6:**
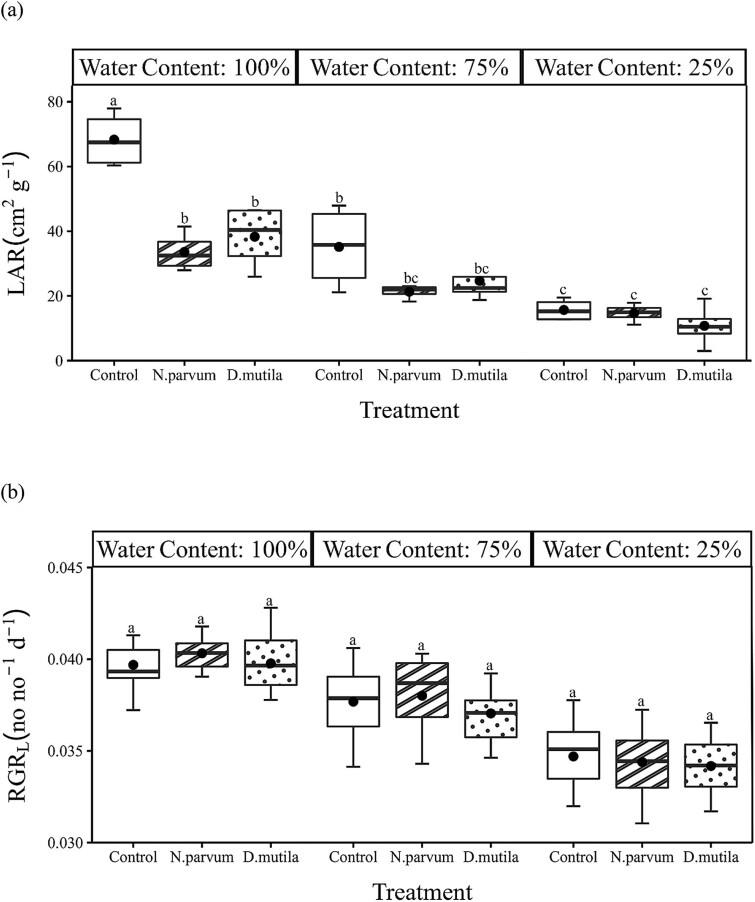
Box and whisker charts showing variation of (a) leaf area ratio (LAR) and (b) relative growth rate of leaves (RGR_L_) for *J. regia* L. in mock-inoculated control (blank boxplot), *N. parvum*-inoculated (stripe boxplot) and *D. mutila*-inoculated (dotted boxplot) trees under 100, 75 and 25% water content treatments. The upper and the lower edges of each box indicate the 75th and 25th percentiles, respectively. Large black dots inside each box plot represent the mean and the horizontal line within each box is the median. Different letters indicate statistically significant differences (Tukey HSD test *P* < 0.05).

In comparison to the control treatment (irrigated at field capacity), deficit irrigation showed significant effects on tree growth and morphology (Tables 2 and 3; Figures 5 and 6). The impact was clearly measurable on LA, LAR, *RGR*_L_ and *M*_L_ (*P* < 0.001) and to a lesser degree *RGR*_D_ (*P* < 0.01) for both pathogens and was also noticeable on a broader range of growth parameters in *N. parvum*-inoculated trees including *M*_P_ (*P* < 0.01), *M*_S_ (*P* < 0.01), and to a lesser extent *M*_R_ (*P* < 0.05) (Tables 3 and 4). There were no significant responses of *RGR*_H_ to any treatment (*P* > 0.05). Trees inoculated with pathogens showed significantly lower LA_plant_ (*P* < 0.01), LAR (*P* < 0.001) and to a lesser degree R/S ratio for *N. parvum*-infected trees (*P* < 0.05) (Tables 3 and 4). Although there was no significant difference in leaf-level gas exchange with pathogen inoculation, reduced LA under pathogen inoculation resulted in a significant reduction of *A*_plant_ (*P* < 0.01; Tables 3 and 4), but only in fully irrigated trees (*P* < 0.001; Figure 5a,b) (*P* < 0.001; Table 2; Figure 5a). Thus, pathogen inoculation effectively reduced the area of leaves that could be supported, but the leaf area that could be deployed under pathogen treatments functioned at similar rates to leaves on control plants.

**Figure 7:**
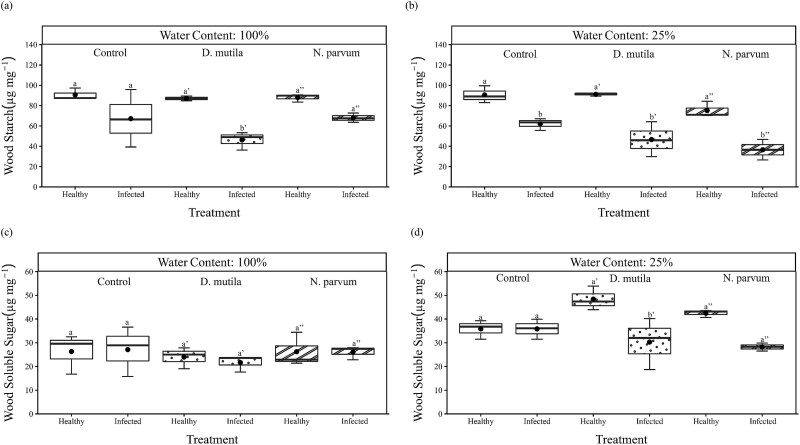
Box and whisker charts showing amounts (μg mg^−1^) of wood starch (a, b) and soluble sugars (c, d), in *J. regia* L. xylem under 100% (a, c) and 25% (b, d) water content and either mock-inoculated (blank boxplot), or inoculated with *D. mutila* (dotted boxplot) or *N. parvum* (stripe boxplot). ‘Healthy’ wood indicates xylem tissue that was not wounded and inoculated whereas ‘infected’ wood indicates xylem tissue in the reaction zone at the vicinity of the wounded and inoculated infection point. The upper and the lower edges of each box indicate the 75th and 25th percentiles, respectively. Large black dots inside each box plot represent the mean, the horizontal line within each box is the median. Different letters indicate statistically significant differences (Tukey HSD test *P* < 0.05).

Our results also showed that deficit irrigation affected the soluble sugar levels in healthy walnut wood (*P* < 0.01; Tables 2 and 3). Soluble sugars were depleted in the wood of infected trees under severe deficit irrigation (Figure 7c,d) although it was only significant for *D. mutila* (*P* < 0.05). Trends of starch depletion were recorded in the reaction zone compared to the healthy wood of well-watered trees, but that depletion significantly worsened for all treatments under severe deficit irrigation (Fig. 7a,b).

## Discussion

The increase of tree mortality induced by drought, pests and diseases has been well documented in forest ecosystems in the context of climate change (Allen et al. 2010, McDowell 2011, Jactel et al. 2012). In arid and semi-arid agroecosystems, precision irrigation tools and strategies have been increasingly deployed to optimize water management under limited water availability. In recent years, there has been a stark increase in disease reports caused by wood pathogens in tree crops and those have been linked to changes of both cultural practices and climate (Gramaje et al. 2016, Batista et al. 2021). *N. parvum* and *D. mutila* are two common pathogens of woody crops and have been widely reported to cause dieback in walnut (Moral et al. 2019, Jimenez Luna et al. 2022). Both taxa have been described as endophytic commensal organisms (Dakin et al. 2010, Blumenstein et al. 2021), that become pathogenic when the affected hosts are under stress (Barradas et al. 2018, Galarneau et al. 2019, Brodde et al. 2023) suggesting that pathogenicity is triggered by environmental cues (Yan et al. 2018). We tested the disease triangle concept to evaluate how pathogen inoculation and infection interacts with reduced water input by mimicking stress-induced deficit irrigation. We expected that in walnut trees, long-term exposure to water stress and wood pathogens would impair host water transport and subsequently host physiology. We also predicted that the interaction of both stresses should have a cumulative effect and exacerbate symptoms. We found that deficit irrigation produced a typical drought-type response of reduced leaf gas exchange and tree growth, but in contrast to expectations, the pathogens had a limited effect that was largely independent of the plant water status.

Previous work has shown that English walnut exhibits a desiccation avoidance strategy, with resources allocated to growing roots in deep soil layers and early stomatal closure to cope with water stress (Gauthier and Jacobs 2011, Sun et al. 2011). Consistent with this finding we found that under deficit irrigation, trees rapidly exhibited a significant decrease in leaf transpiration and stomatal conductance (Cochard et al. 2002). The leaf rachis is the most vulnerable organ along the water transport pathway under water stress, and the tree host regulates stomatal closure to maintain Ψ_Leaf_ above −1.6 MPa to prevent xylem failure (Cochard et al. 2002). These conditions were met at the end of the experiment in severely drought stressed trees. Tyree et al. (1993) showed that, as part of its desiccation avoidance response, walnut trees senesced leaves after being exposed to five consecutive days of drought, once tissues reach around −1.9 MPa or less (Tyree et al. 1993). This threshold was never reached under our conditions, but trees expressed symptoms at Ψ_Leaf_ − 1.6 MPa after a prolonged time under drought conditions (20 weeks under 75% deficit irrigation). Our data also recorded additional morphological and growth responses to deficit irrigation, especially reduced total leaf area and leaf area ratio. Those effects were compounded by a reduced leaf gas exchange per unit leaf area causing a significant reduction in photosynthetic and evaporative surface area at the whole plant scale. As expected, the drought conditions imposed during dormancy negatively affected tree growth resulting in the reduction in leaf, branch and root biomass (Gauthier and Jacobs 2011, Sun et al. 2011, Liu et al. 2019). We were unable to evaluate the growth response of roots to mediate the effects of deficit irrigation because we used potted 2-year old trees with an established root system that was constrained for growth.

Pathogen infection could have also reduced stem xylem water potential because of vessel occlusion (Venturas et al. 2014, Bortolami et al. 2021). However, the lack of significant interaction with the irrigation treatments (Tables 2 and 3) suggests that the pathogen-induced vessel occlusion affected plant hydraulic functions in the same capacity under the different water stress levels. These results are consistent with similar necrotic lesion length in all water treatments. Whole-plant photosynthesis was also strongly reduced by pathogens which was driven by LAR because the leaf-level photosynthetic gas exchange rates showed no significant effects in response to the pathogen treatment. Our data confirmed that pathogen inoculations had different effects on LAR depending on the irrigation regime. This apparent reduction in the number of leaves that can be supported with reduced water transport is also consistent with vascular pathogen blockage. One of the most striking results of our study was that walnut responded strongly to deficit irrigation, but responses to pathogen infection were minimal or non-existent under deficit irrigation. This is in contrast with several studies showing an increase in symptom severity with stress (Schoeneweiss 1983, Mullen et al. 1991, Desprez-Loustau et al. 2006, Galarneau et al. 2019). Jactel et al. (2012) proposed that the negative effects of wood endophytic pathogens are only significant above a water stress threshold of 30%, as determined by the ratio between predawn leaf water potential and *P*_50_ value (50% of hydraulic conductivity loss due to xylem cavitation). The *P*_50_ of walnut is around −2 MPa (Jinagool et al. 2018) and thus the critical water stress threshold was reached when Ψ_PD_ equaled −0.6 MPa. This threshold was recorded in the 75% deficit irrigation treatment between Julian dates 120 and 140 and again after Julian date 180, when Ψ_MD_ also reached −1.6 MPa and leaves started to turn yellow and dropped. The sequential order of drought onset in relation to pathogen infection has also been proposed as important factors in disease outcome (Caldeira 2019, Hossain et al. 2019). The theory is that initial drought-stress conditions predispose the host and increases its vulnerability to the pathogen. In our experiment, both abiotic and biotic stresses were applied at the same time on dormant trees and resulted in similar necrotic lesion length across water-stress treatments. Our results did not verify that *N. parvum* was more virulent than *D. mutila* even under drought stress (Jimenez Luna et al. 2022), perhaps because trees maintained hydraulic function levels above the critical water stress threshold. Another possible reason is that Koch’s postulates for wood decay fungi is commonly verified on young tree branches and species of *Neofusicoccum* have been shown to progress slower in older lignified tissues (Agustí-Brisach et al. 2019, Tennakoon et al. 2022). Thus, it may require a longer incubation time to distinguish levels of virulence between taxonomic groups when using tree trunks.

One possible explanation for pathogen inoculation responses being strongest in fully irrigated trees is that reduced water supply limits the function of the pathogen (Leisner et al. 2023). There is ample evidence that high water availability is beneficial to the activity of many fungal pathogens, and that drought could alter the balance of plant-pathogen interactions (Chakraborty and Newton 2011, Ghelardini et al. 2016, Velasquez et al. 2018). However, it is also important to consider that most fungal pathogens display a physiological plasticity and can function at water potentials below the minimum for growth of their host (Desprez-Loustau et al. 2006). Another possible explanation for the muted response to pathogen infection during drought is that drought reduces a wide range of physiological activity that leaves little room for discernable effects caused by the pathogen. This mechanism is based on the idea that the effects of xylem vessel blockage induced by fungal pathogens are negligible when there is already some extended degree of xylem vessel cavitation with minimal water to transport. This suggests that water deficit has the same effect on tree water status regardless of the pathogen, at least in a scenario of a mild disease levels. Additional experiments will need to be performed under severe pathogen infection where most of the host vasculature has already lost hydraulic functions due to occlusion. In this scenario, we would predict that drought would cavitate the remaining active xylem vessels and likely worsen symptom severity.

We also measured an effect of both drought and pathogen on the host pool of NSC. The effect of irrigation on wood soluble sugars and depletion of starch in the reaction zone suggested that starch was being converted to sugars to meet the carbohydrates demand for osmotic adjustment and metabolic energy (Nardini et al. 2011). Starch was also depleted in the necrotic wood of infected trees indicating that resources were allocated by the host for cell wall reinforcement against the invading pathogens or that it was consumed by the fungus during pathogenesis (Rolshausen et al. 2008, Pouzoulet et al. 2017, Pouzoulet et al. 2021). It would be interesting to capture how the NSC pool is affected when a synergistic effect is observed between drought and pathogen virulence that yield larger necrotic lesions.

Overall, our study provides a better understanding of host biotic and abiotic interactions in an agricultural tree crop and illustrates the complex balance between pathogen infection and environmental conditions as described in the disease triangle framework. Together, our results suggest that drought had a much deeper impact on a broad range of physiological functions regardless of fungal infection. In contrast, the range of effects induced by fungal infection was limited and was only measurable when trees where fully irrigated.

## Data Availability

Data and R-Script can be requested from the first author.
